# Peroral endoscopic myotomy for the treatment of achalasia in the Unified Healthcare System (SUS): results of a short-term

**DOI:** 10.1590/0100-6991e-20223244-en

**Published:** 2022-12-02

**Authors:** IGOR RABELO DE FRANÇA, EDUARDA AUGUSTA DE LUCENA CALDAS, MARCELLA FERREIRA BARROS, JOSE TARCÍSIO DIAS DA SILVA, JOÃO PAULO PONTUAL, ÁLVARO A. B. FERRAZ

**Affiliations:** 1 - Universidade Federal de Pernambuco, Departamento de Cirurgia - Recife - PE - Brasil; 2 - Universidade Federal de Pernambuco, Curso de Medicina - Recife - PE - Brasil

**Keywords:** Esophageal Achalasia, Esophageal Motility Disorders, Fundoplication, Acalasia Esofágica, Transtornos da Motilidade Esofágica, Fundoplicatura

## Abstract

**Introduction:**

achalasia is a chronic disease. Since there is no curative treatment, diagnosed patients have pharmacological and/or surgical techniques available, aimed at minimizing the condition. POEM appears as a promising new type of palliative treatment with good rates of symptom improvement.

**Objective:**

evaluate the profile of POEM at the Clinical Hospital of the Federal University of Pernambuco (HC - UFPE) and correlate it with the world scenario.

**Methods:**

data collection was performed retrospectively from September 2017 to October 2019 with all patients undergoing POEM at the HC - UFPE. Sociodemographic, clinical, and hospital variables were evaluated before and three months after the procedure.

**Results:**

of 27 patients (52.41 ± 19.24 years old) who underwent the procedure, 66.7% had idiopathic etiology and 33.3% had etiology secondary to Chagas disease. 48% patients underwent previous procedures, of which seven used some type of medication for symptom control, two underwent pneumatic endoscopic dilation, and four underwent Heller cardiomyotomy with partial fundoplication. 62.5% of the evaluated patients had type II achalasia before the procedure. Seven (25.9%) patients presented the following adverse events: four presented bleeding, two pneumoperitoneum, and one both complications, all being treated conservatively. The Eckardt score reduced from 8.37 ± 1.45 to 0.85 ± 1.06 (p-value <0.001).

**Conclusion:**

clinical improvement of symptoms and the patient profile followed the worldwide trend, with emphasis on the etiology secondary to Chagas disease, endemic in Brazil. Gastroesophageal reflux remains the main post-operative symptom.

## RESUMO

**Introdução:**

a acalasia é uma doença crônica. Por não haver tratamento curativo, os pacientes diagnosticados dispõem de técnicas farmacológicas e/ou cirúrgicas, visando minimizar o quadro. A POEM surge como um novo tipo de tratamento paliativo promissor com boas taxas de melhora dos sintomas.

**Objetivo:**

avaliar o perfil das POEM realizadas no Hospital das Clínicas da Universidade Federal de Pernambuco (HC - UFPE) e correlacioná-lo com o cenário mundial.

**Métodos:**

a coleta de dados foi realizada retrospectivamente no período de setembro de 2017 a outubro de 2019 com todos os pacientes submetidos a POEM no HC - UFPE. Variáveis sociodemográficas, clínicas e hospitalares foram avaliadas antes e três meses após o procedimento.

**Resultados:**

total de 27 pacientes (52,41 ± 19,24 anos) que realizaram o procedimento, 66,7% com etiologia idiopática e 33,3% com etiologia secundária à doença de Chagas. 48% tinham sido submetidos a procedimentos prévios, dos quais sete usaram algum tipo de medicamento para controle dos sintomas, dois foram submetidos à dilatação endoscópica pneumática e quatro à cardiomiotomia a Heller com fundoplicatura parcial. 62,5% dos pacientes avaliados tinham acalasia tipo ii antes do procedimento. Sete (25,9%) apresentaram eventos adversos: quatro sangramentos, dois pneumoperitônio e um ambas as complicações, todos foram tratados de forma conservadora. O escore de Eckardt reduziu de 8,37 ± 1,45 para 0,85 ± 1,06 (valor de p<0,001).

**Conclusão:**

melhora clínica e o perfil dos pacientes acompanhou a tendência mundial, destaque para a etiologia secundária à doença de Chagas, endêmica no Brasil. O refluxo gastroesofágico continua sendo o principal sintoma pós-operatório.

## INTRODUCTION

Achalasia is a primary disorder of the esophagus and has a worldwide incidence of 1.8 to 12.6 per 100,000 individuals. Its main etiologies are idiopathic and secondary to Chagas disease^1^ ^,^ ^2^. The clinical picture is manifested with progressive dysphagia, weight loss, regurgitation, heartburn, retrosternal pain, and respiratory symptoms. 

The Eckardt score is a self-report assessment tool used to objectively quantify the symptoms of the disease, being used as the main parameter to evaluate treatment effectiveness^3^ ^-^ ^7^. 

The gold standard for its diagnosis is esophageal manometry. However, the use of other complementary exams is necessary, such as upper digestive endoscopy and barium esophagogram, in order to rule out anatomical obstructions, malignancy, gastroesophageal reflux disease, and complications of achalasia itself^7^.

Since there is no curative treatment, patients diagnosed with this pathology use pharmacological and/or surgical techniques in order to minimize the clinical condition. Botulinum toxin injection, endoscopic pneumatic dilation and Heller cardiomyotomy with partial fundoplication, which is the gold standard in therapy, are among the options with the best success rates^7^ ^,^ ^8^. 

In this context, a new surgical approach was developed by Inoue and collaborators, the peroral endoscopic myotomy (POEM)^9^ ^,^ ^10^. The POEM, besides being a safe technique, minimally invasive, and with success rates equivalent to surgery, the existence of previous procedures, both clinical or surgical, do not contraindicate its performance^8^ ^,^ ^11^. This study evaluates the etiological, sociodemographic, clinical and hospital profile of patients, besides their results immediately and three months after undergoing POEM at the Clinical Hospital of the Federal University of Pernambuco (HC - UFPE).

## METHODS

Data collection was conducted from September 2017 to October 2019 at the Clinical Hospital of the Federal University of Pernambuco through analysis of medical records of patients undergoing POEM.

### Patients

All patients who underwent the procedure in the period were included in the survey. The sample included 28 patients in a non-random manner, with the volunteers being selected consecutively. The exclusion criteria of this study were, as follows: post-operative period not performed at HC - UFPE, refusal to participate in the collection, unsuccessful contact or incomplete medical records. Only one patient was dismissed from the sample for not performing the post-operative follow-up at HC - UFPE, resulting in a total of 27 patients in this study. For statistical analysis correlating the degree of achalasia by the esophagogram, a total of 24 patients were used, three patients were excluded from the correlation because did not have preoperative esophagograms in their medical records, only previous digestive endoscopy. This study was submitted to the Ethics and Research Committee of HC - UFPE was approved CAAE No. 27695320.4.0000.8807.

### Procedures

**Figure 1:**
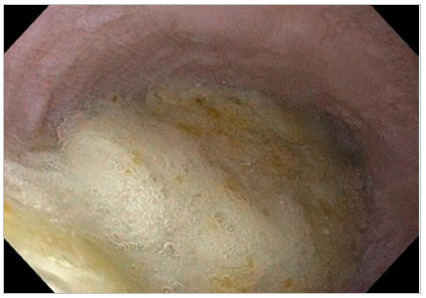
Stasis is present in most POEMs. Orotracheal intubation should consider the high risk of bronchoaspiration.

**Figure 2:**
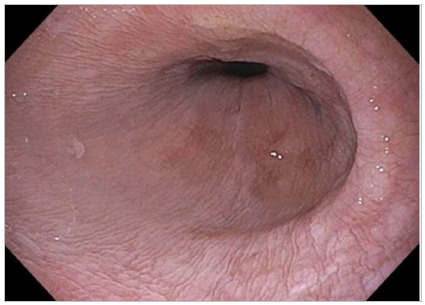
Dilated esophagus showing edematous mucosa and effacement of the vasculature.

**Figure 3:**
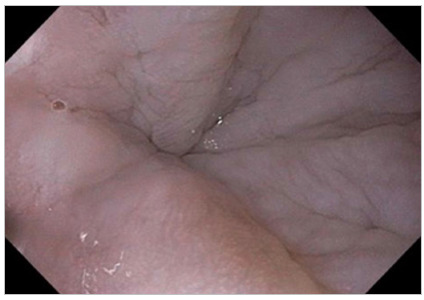
Hypertonic cardia of difficult transposition.

**Figure 4:**
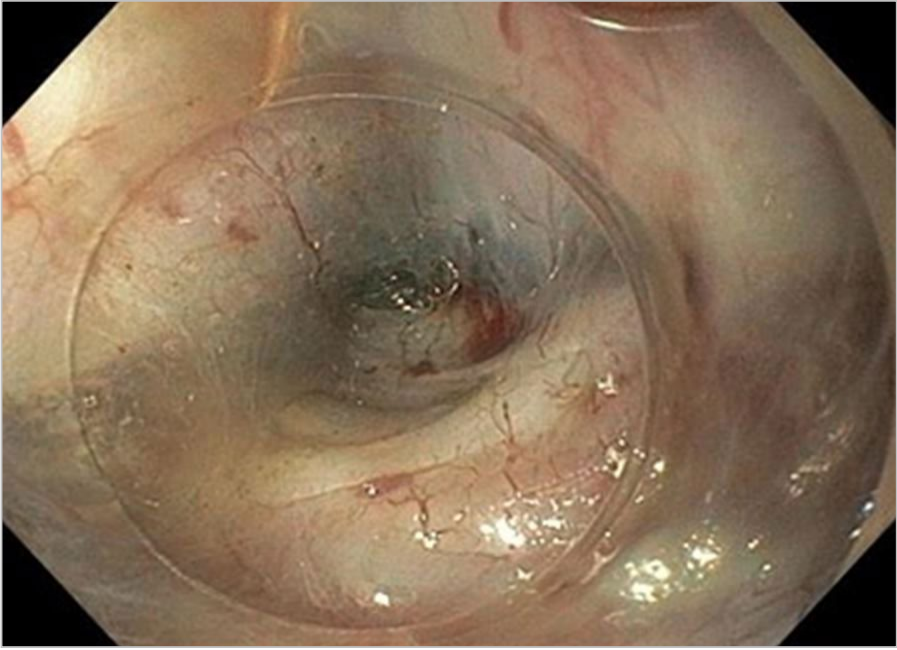
Submucosal tunneling close to the muscle.

**Figure 5:**
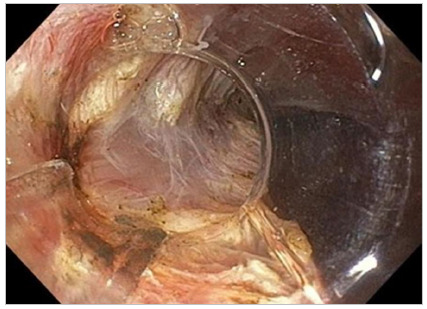
Myotomy of the circular layer with preservation of the longitudinal layer.

**Figure 6:**
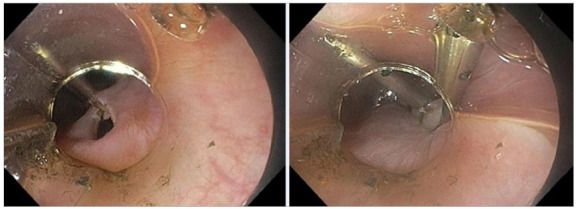
Clip closure of the mucosotomy.

All procedures were performed by the same specialist with extensive experience in interventional endoscopy. Under CO_2_ insufflation, the tunnel was started with mucosotomy approximately 10cm away from the cardia, located at the five o’clock position, with the patient in supine position and under general anesthesia. The submucosal plane close to the muscle was dissected until reaching 2.0cm below the cardia, followed by myotomy of the circular muscle layer, initiated 2.0cm below the mucosotomy. Sectioning of the longitudinal esophageal musculature was necessary in cases of fibrosis with difficulty in separating the elements or in very intense hypertonias of the lower esophageal sphincter. Myotomy included an inch or two from the stomach muscles. The tunnel was sealed with the closure of the mucosotomy by endoscopic clips (Figures 1 to 6).

The collected data included sociodemographic, clinical, and hospital variables. Symptoms were evaluated using the Eckardt score in the preoperative period and at the return visit (two to three months later), in which any complications and symptoms were evaluated. This score consists of a self-reported evaluation used to quantify the main symptoms pre- and post-treatment. Active achalasia is considered when the score is higher than three points and treatment effectiveness is considered when the score is lower and/or equal to three^5^ ^-^ ^7^. Regarding the procedure, the following data were evaluated: size of the tunnel and myotomy performed, partial or total section of the circular fibers, and complications during the procedure.

### Statistics

The softwares SPSS 13.0 (Statistical Package for the Social Sciences) for Windows and Excel 2010 were used. All tests were applied with 95% confidence intervals and the results were presented in a table with their respective absolute and relative frequencies. Categorical variables were presented as percentages and analyzed using Fisher’s exact test. Numerical variables are represented by measures of central tendency and measures of dispersion. The normality test of Shapiro-Wilk was used for quantitative variables (n<30) and the paired t-test (normal distribution) and Wilcoxon test (non-normal) were used for the test between paired groups.

## RESULTS

POEM was performed in a total of 27 patients (13 women - 48.9% and 14 men - 51.1%, with mean age of 52.41 ± 19.24 years old). Clinical and epidemiological data were summarized in Table 1. The predominant etiology was idiopatic, with 18 patients (66.7%), with esophagopathy secondary to Chagas disease being the second most predominant etiology with nine patients (33.3%). Thirteen patients (48%) who underwent POEM had already undergone some type of previous procedure. Out of these, seven (53.8%) used some type of medication for symptom control, two (15.4%) underwent pneumatic endoscopic dilation, and four (30.8%) underwent Heller cardiomyotomy with partial fundoplication, most of them underwent POEM in Rezende and Mascarenhas’ type II (58%).

**Table 1:** Clinical and epidemiological data of patients who underwent POEM.

Variables	n	%
Sex		
Male	14	51.9
Female	13	48.1
Comorbidities		
Arterial Hypertesion	7	25.9
Diabetes Mellitus	1	3.7
Smoking	2	7.4
Alcoholism	7	25.9
Variables	n	%
Other comorbidities	7	25.9
Previous treatment		
Yes	13	48.1
No	14	51.9
Type of previous treatment		
Medicative	7	53.8
Endoscopic dilation	2	15.4
Heller	4	30.8
Etiology		
Idiopathic	18	66.7
Chagas’ disease	9	33.3

According to Rezende and Mascarenhas’s classification, there was a predominance of type II (62.5%), followed by type III (21%), type I (12.5%), and only one patient (4%) had type IV before the procedure. Regarding the etiology, idiopathic and chagasic, most had type II, corresponding to 75% and 56.2%, respectively (Table 4).

Among the 27 patients who underwent POEM, 14 (51.9%) underwent sectioning only of the circular fibers and 13 (48.1%) required total sectioning of the fibers due to technical difficulty or the need for greater relief in the lower esophageal sphincter pressure. Details of the procedure were summarized in Table 2. Mean tunnel length was 12.54 ± 2.09cm and myotomy length was 9.46 ± 1.96cm, with a 1.37 ± 0.51cm section being performed after the cardia. 

**Table 2:** Data of the procedures performed.

Variables	n	%
Complications in the procedure		
Yes	7	25.9
No	20	74.1
Type of complication in the procedure		
Bleeding	4	57.1
Pneumoperitoneum	2	28.6
Bleeding and Pneumoperitoneum	1	14.3
Tissue Section		
Variables	n	%
Circular	14	51.9
Total	13	48.1
Complications or symptoms in the follow up		
Present	13	48.1
Absent	14	51.9
Type of complications or symptomatology		
Reflux	9	69.2
Dysphagia for solids	4	30.8
	Mean ± SD	Minimum - Maximum
Age	52.41 ± 19.24	11.00 - 85.00
Tunnel Size (cm)	12.54 ± 2.09	8.00 - 16.00
Myotomy size (cm)	9.46 ± 1.96	5.00 - 13.00
Gastric component (cm)	1.37 ± 0.51	1.00 - 2.50

Adverse events were observed in seven (25.9%) patients. Out of these, four (57.1%) presented controlled bleeding, two (28.6%) pneumoperitoneum and one (14.3%) both complications. Most of the operative complications occurred in patients with type II achalasia (71%), which may be related to the fact that this group comprised the largest sample. The other 28% of patients who had complications belonged to types I and III, corresponding to one patient in each (Table 4). Nabi et al.^12^ classified adverse events as major and minor according to their classification. Thus, all events observed in this study were minor. The surgeries were concluded without interruption and all patients who had adverse intraoperative events were treated during the procedure itself, without the need for additional interventions. None of the adverse events had late clinical repercussions.

**Figure 7:**
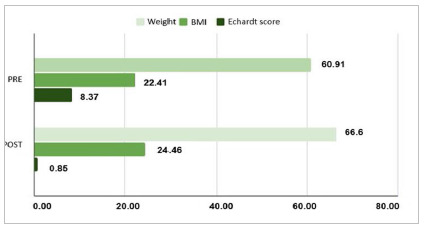
Comparison between pre- and post- POEM parameters.

The 27 patients were followed up on an outpatient basis, on average three months after the procedure. Reduction in the Eckardt score (Table 3) from 8.37 (± 1.45) to 0.85 (± 1.06) was observed from the pre- to post-operative consultation. Increases of mean body weight from 60.91 (± 18.91kg) to 66.60 (± 19.03kg) and of BMI from 22.41 (± 5.39kg/m²) to 24.46 (± 5.10kg/m²) were observed after POEM, as shown in Figure 7, with significance of p-value <0.001. In the first three months, 13 (48.1%) patients presented some type of complication or recurrent symptomatology, out of which nine patients presented gastroesophageal reflux and four dysphagia for solids, representing 33.3% and 14.81% of the total of patients, respectively.

**Table 3:** Eckardt score before and after treatment with POEM.

	Moments		
Variables	Pre	Post	p-value
	n (%)	n (%)	
Weight loss			
0	2 (7.4)	24 (88.9)	< 0.001 *
1	8 (29.6)	2 (7.4)	
2	6 (22.2)	1 (3.7)	
3	11 (40.7)	0 (0.0)	
Dysphagia			
0	0 (0.0)	18 (66.7)	< 0.001 *
1	0 (0.0)	8 (29.6)	
2	2 (7.4)	1 (3.7)	
3	25 (92.6)	0 (0.0)	
Regurgitation			
0	1 (3.7)	22 (81.5)	< 0.001 *
1	2 (7.4)	5 (18.5)	
2	7 (25.9)	0 (0.0)	
3	17 (63.0)	0 (0.0)	
Retrosternal pain			
0	11 (40.8)	24 (88.9)	0.001 *
1	9 (33.3)	2 (7.4)	
2	3 (11.1)	0 (0.0)	
3	4 (14.8)	1 (3.7)	

(*)Fisher’s exact test.

**Table 4:** Degree of achalasia and its correlations.

Variables	n	%
Degree of achalasia		
Type I	3	12.5
Type II	15	62.5
Type III	5	21
Type IV	1	4
Variables	n	%
Degree of achalasia in patients with Chagas' etiology		
Type I	0	0
Type II	6	75
Type III	1	12.5
Type IV	1	12.5
Degree of achalasia in patients with Idiopathic’s etiology		
Type I	3	18.7
Type II	9	56.2
Type III	4	4
Type IV	0	0
Postoperative complications according to the degree of achalasia		
Type I	1	14
Type II	5	71
Type III	1	14
Type IV	0	0
Degree of achalasia after previous treatment		
Type I	3	25
Type II	7	58.3
Type III	1	8.3
Type IV	1	8.3

## DISCUSSION

Achalasia is a primary esophageal motor disorder whose incidence has remained stable in recent years, ranging from 1.8 to 12.6 per 100,000 individuals, having similar prevalence in most countries^1^ ^,^ ^2^. Since it is a primary disease, the main cause is idiopathic etiology. However, the clinical picture can mimic other conditions, such as esophagopathy secondary to Chagas disease. 

Since it is a disease without a curative intervention, patients resort to palliative therapies to reduce clinical symptoms. Some techniques have good success rates, including botulinum toxin injection, endoscopic pneumatic dilation, and Heller cardiomyotomy with partial fundoplication, which is the gold standard in therapy^7^ ^,^ ^8^. A new technique developed by Inoue and collaborators, called peroral endoscopic myotomy, was described in 2010^9^ ^,^ ^10^. In the present study, sociodemographic, clinical, and hospital variables, besides those related to the POEM technique were evaluated at the HC-UFPE. 

The main etiology of achalasia was idiopathic, corresponding to a total of 18 (66.7%) patients, followed by esophagopathy secondary to Chagas disease, with nine (33.3%) patients with positive serology, following the world trend^2^ ^,^ ^8^. The disease’s etiology was defined through epidemiology and positivity Chagas’ disease serology; high prevalence of Chagas’ disease was already expected, since the northeast of Brazil is still an endemic region despite the combat measures conducted in the last century. In addition, it is known that approximately 15-20% of the population with the disease develops gastrointestinal changes, such as megacolon, and 20-30% after ten years of disease may develop cardiologic alterations, however, as it was not the objective of the study, these alterations were not included in the current study. In patients whose serology was negative, the disease was defined as idiopathic, even though it is known that there are external factors that may influence the development of achalasia, it was not possible to associate such factors due to lack of information in the medical records^3^ ^,^ ^4^ ^,^ ^13^ ^,^ ^14^. 

According to the last guideline, the gold-standard to determine the degree and severity of achalasia is the Chicago’s Classification using a high-resolution manometry, that is not available in the public health system in Pernambuco. This classification was created in 2011 by the International High Resolution Manometry Working Group^15^ as a way of classifying the degree of esophageal diseases through manometry. For that reason, it was decided to use the Rezende e Mascarenhas’ Classification, which uses the esophagogram to define the severity of achalasia. The classification determines four degrees: Type I (unchanged caliber, discreet contrast retention, <4cm); Type II (moderate increase of caliber, contrast retention and tertiary waves, 4-7cm); Type III (large increase of caliber and hypotonia, 7-10cm); and Type IV (dolico-megaesophagus, >10cm)^16^. In a total of 27 patients included in the study, 24 patients had data from these exams in their medical records to calculate the correlation, three patients underwent POEM with only previous endoscopic exams proffing achalasia. According to the latest recommendations^17^ ^,^ ^18^, POEM can be indicated for treatment in all stages of the disease, especially for more advanced cases being a less invasive procedure. In our study, 62.5% presented with type II achalasia, 25% with types III or IV, and only three patients (12.5%) in type I. It is worth emphasizing that many patients in the public health system have difficulty to access the most modern therapies, many of them presenting for treatment in more advanced stages of the disease, almost 90% of the sample already had moderate esophageal dilation (> or =4cm) with contrast retention and motility alteration when allocated to the study.

Regardless of the degree of disease involvement, patients of this study were followed up on an outpatient basis at the two following moments: preoperatively and on average two to three months after the procedure. During the follow up, the Eckardt score was applied before and after the POEM, obtaining an average preoperative score of 8.37 (± 1.45) and post-operative of 0.85 (± 1.06). The score is used to evaluate the repercussions of the procedure, with a favorable result being considered if the score is lower and/or equal to three^8^. In the present study, 26 patients had a score compatible with therapeutic success. Only one patient had an Eckardt score greater than three after the procedure and it was a suspicion of esophageal spastic disorder according to pre-procedure endoscopy, who has esophagogram type I.

Despite being limited to assessing short-term follow-up, other recently published studies with long-term follow-up demonstrate that the satisfaction with the technique was reported by more than 90% of the patients followed^19^ ^,^ ^20^. This fact suggests that POEM is a good long-term option for treating patients with achalasia, guaranteeing good results.

Six patients followed had already been treated previously, either by surgical or endoscopic treatment, and showed no significant difference in the post-POEM Eckhart Score when compared to patients who were not treated previously, which is in agreement with what Tan et al. and Sanaka et al. stated in their most recent studies^21^ ^,^ ^22^. 

Among Eckardt score variables, dysphagia alone contributed most significantly to a higher score, followed by regurgitation. Progressive dysphagia at all meals was reported by 92.6% of patients and regurgitation by 63%. These rates are similar to those observed by Boeckxstaens et al.^3^, who reported dysphagia as the main symptom of achalasia, affecting more than 90% of patients, with regurgitation ranging between 76-91%. Although the Eckardt score is a widely used evaluation tool, with a good level of recommendation by the last guideline for the treatment of achalasia^8^, the study by Taft et al.^6^ showed that the weight loss and retrosternal pain variables can decrease the reliability and validity of the score.

The main criticism of this endoscopic technique in the world literature is related to the high rates of gastroesophageal reflux disease when compared with other treatment method^9^ ^,^ ^11^ ^-^ ^14^ ^,^ ^23^ ^-^ ^24^. During short-term follow up, it was observed that 48.1% of patients reported some recurrent symptoms. Symptoms related to gastroesophageal reflux were self-reported by 33.3% of the patients, standing out as a major challenge in the post-operative period. However, the presence of gastroesophageal reflux was identified through the patient’s clinical, thereby, the rate of gastroesophageal reflux observed in the study may be underestimated, since diagnostic tests were not performed after symptoms. Those patients who do not respond to the POEM, others palliative alternatives can be used, as pneumatic dilation to older techniques such as esophagectomy to Thal-Hatafuku^25^ ^,^ ^26^.

Although relevant perioperative adverse events are uncommon, 25.9% had minor complications, such as controlled bleeding (14.8% of patients) and pneumoperitoneum (7.4%), with one patient having both, most of them were evidenced in type II patients, probably due to a larger sampling of this group. All adverse events were considered minor according to the classification proposed by Nabi et al.^12^ and were treated conservatively during the procedure, without immediate or delayed clinical repercussions. 

Despite the above, our work had some limitations the lack of data in the medical records difficulted clarify previous history and etiology. The difficulty of access by the public health system to the gold standard, esophageal manometry, for diagnosis and degree of severity. Many patients lost follow up due to the fact that the Hospital had become a center for the treatment of COVID-19, compromising medium and long-term follow-up. In addition, Hospital das Clínicas is characterized as a tertiary care unit within the Brazilian public health system and only one professional in this service is qualified to perform POEM.

## CONCLUSION

The peroral endoscopic myotomy performed during this study showed excellent results, following the worldwide trend, with improvement in the clinical symptoms of the patients who underwent the procedure. POEM has become a good therapeutic option and more studies are necessary for a better evaluation of patients and maintenance of medium- and long-term follow up.

## References

[1] Pressman A, Behar J (2017). Etiology and pathogenesis of idiopathic achalasia. J Clin Gastroenterol.

[2] Sadowski DC, Ackah F, Jiang B, Svenson LW (2010). Achalasia: Incidence, prevalence and survival. A population-based study. NeurogastroenterolMotil.

[3] Boeckxstaens GE, Zaninotto G, Richter JE (2014). Achalasia. Lancet.

[4] Pinazo MJ, Lacima G, Elizalde JI, Posada EJ, Gimeno F, Aldasoro E (2014). Characterization of digestive involvement in patients with chronic T cruzi infection in Barcelona, Spain. PLoS Negl Trop Dis.

[5] Shemmeri E, Aye RW, Farivar AS, Bograd AJ, Louie B E (2019). Use of a report card to evaluate outcomes of achalasia surgery beyond the Eckardt score. Surg Endosc.

[6] Taft TH, Carlson DA, Triggs J, Craft J, Starkey K, Yadlapati R (2018). Symptom score as a measure of achalasia severity. Neurogastroenterol Motil.

[7] Schlottmann F, Patti MG (2018). Esophageal achalasia: current diagnosis and treatment. Expert Rev GastroenterolHepatol.

[8] Zaninotto G. (2018). The 2018 ISDE Achalasia guidelines. Dis Esop.

[9] Inoue H, Sato H, Ikeda H, Onimaru M (2015). Per-oral endoscopic myotomy a series of 500 Patients. J Am Coll Surg.

[10] Vaezi MF, Felix VN, Penagini R, Mauro A, Guimar E, Moura H. (2016). Achalasia from diagnosis to management. Ann N Y Acad Sci.

[11] Zaninotto G, Bennett C, Boeckxstaens G, Costantini M, Ferguson MK, Pandolfino JE (2018). The 2018 ISDE achalasia guidelines. Diseases of the Esophagus. Dis Esophagus.

[12] Nabi Z, Reddy DN, Ramchandani M Adverse events during and after per-oral endoscopic myotomy prevention, diagnosis, and management. Gastrointest Endosc.

[13] Dantas RO (2003). Comparison between idiopathic achalasia and achalasia caused by Chagas' disease a review on the publications about the subject. Arq Gastroenterol.

[14] Stanaway JD, Roth G (2015). The Burden of Chagas Disease. Global Heart.

[15] Bredenoord AJ, Fox M, Kahrilas PJ, Pandolfino JE, Schwizer W, Smout AJ (2012). International High Resolution Manometry Working Group. Chicago classification criteria of esophageal motility disorders defined in high resolution esophageal pressure topography. Neurogastroenterol Motil.

[16] Rezende JM (1982). Classificação radiológica do megaesôfago. Rev Goiana Med.

[17] Vaezi M, Pandolfino J, Yadlapati R, Greer K, Kavitt R (2020). ACG Clinical Guidelines Diagnosis and Management of Achalasia. Am J Gastroenterol.

[18] Jung H, Hong S, Lee O, Pandolfino J, Park H, Miwa H (2020). 2019 Seoul Consensus on Esophageal Achalasia Guidelines. J Neurogastroenterol Motil.

[19] Modayil RJ, Zhang X, Rothberg B, Kollarus M, Galibov I, Peller H (2021). Peroral endoscopic myotomy 10-year outcomes from a large, single-center U.S. series with high follow-up completion and comprehensive analysis of long-term efficacy, safety, objective GERD, and endoscopic functional luminal assessment. Gastrointest Endosc.

[20] McKay S, Dunst C, Sharata A, Fletcher R, Reavis K, Bradley D (2021). POEM clinical outcomes beyond 5 years. Surgical Endoscopy Surg Endosc.

[21] Tan S, Zhong C, Ren Y, Luo X, Xu J, Fu X (2021). Efficacy and Safety of Peroral Endoscopic Myotomy in Achalasia Patients with Failed Previous Intervention A Systematic Review and Meta-Analysis. Gut Liver.

[22] Sanaka MR, Khoudari G, Parikh M, Thota PN, Lopez R, Gupta N (2021). Peroral endoscopic myotomy is highly effective for achalasia patients with recurrent symptoms after pneumatic dilatation. Surg Endosc.

[23] Kumbhari V, Familiari P, Bjerregaard N, Pioche M, Jones E, Ko W (2017). Gastroesophageal reflux after peroral endoscopic myotomy a multicenter case-control study. Endoscopy.

[24] Hungness ES, Sternbach JM, Teitelbaum EN, Kahrilas PJ, Pandolfino JE, Soper NJ (2016). Per-oral Endoscopic Myotomy (POEM) After the Learning Curve. Ann Surg.

[25] Ferraz AA, Da Nóbrega BG, Mathias CA, Bacelar TS, Lima FE, Ferraz EM (2001). Late results on the surgical treatment of Chagasic megaesophagus with the Thal-Hatafuku procedure. J Am Coll Surg.

[26] Trevenzol Hélio Ponciano (2010). Recurrence after cardiomyotomy: diagnosis, technical options and results. ABCD Arq Bras Cir Dig.

